# Anticancer Activity of Marine Sponge *Hyrtios* sp. Extract in Human Colorectal Carcinoma RKO Cells with Different p53 Status

**DOI:** 10.1155/2014/413575

**Published:** 2014-08-27

**Authors:** Hyun Kyung Lim, Woori Bae, Hyi-Seung Lee, Joohee Jung

**Affiliations:** ^1^College of Pharmacy, Duksung Women's University, No. 33, 144-gil, Samyang-ro, Dobong-gu, Seoul 132-714, Republic of Korea; ^2^Korea Institute of Ocean Science & Technology, 787 Haeanro, Ansan 426-744, Republic of Korea

## Abstract

Drug development using marine bioresources is limited even though the ocean occupies about 70% of the earth and contains a large number of biological materials. From the screening test of the marine sponge extracts, we found *Hyrtios* sp. sponge collected from Chuuk island, Micronesia. In this study, the *Hyrtios* sp. extract was examined for anticancer activity against human colorectal carcinoma RKO cells that are wildtype for p53 and RKO-E6 that are p53 defective. The *Hyrtios* sp. extract dose-dependently inhibited viability in both cell lines. Multinucleation as an indication of mitotic catastrophe was also observed. Cytotoxicity tests gave significantly different results for RKO and RKO-E6 cells after 48 h exposure to *Hyrtios* sp. extract. In RKO cells treated with *Hyrtios* sp. extract, cell death occurred by induction of p53 and p21 proteins. In p53-defective RKO-E6 cells, *Hyrtios* sp. extract decreased expression of JNK protein and increased p21 protein. These results indicate that *Hyrtios* sp. extract induced apoptosis *via* different pathways depending on p53 status and could be a good natural product for developing new anticancer drugs.

## 1. Introduction

**Figure 1:**
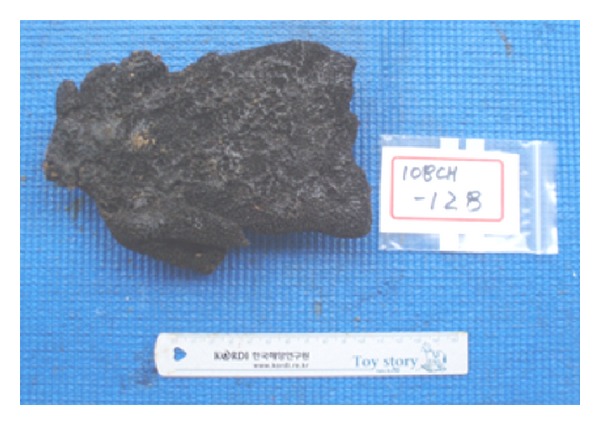
Morphology of* Hyrtios* sp. specimen before methanol extraction.

The ocean occupies about 70% of the earth and contains a huge number of marine organisms. Collection and identification of marine organisms were difficult for researchers and drug developers, but marine resources are still attractive for the application of pharmaceutical fields. Among marine resources, marine sponges are known to have about 15,000 species worldwide [1]. Marine sponges take in nutritious through body pores and produce secondary metabolites with bioactivity. In our ongoing research, we investigated the bioactivity of marine sponges before classifying and isolating their active compounds. Crude extracts were made from marine sponges collected from the Chuuk islands in Micronesia and investigated for anticancer effect. Screening tests identified some specimens with anticancer effects. One of the specimens was identified as* Hyrtios* sp. (Figure 1). Recently,* Hyrtios* sp. was reported to have cytotoxic [2, 3] and antioxidant activities [4]. Several* Hyrtios* metabolites [5, 6] and active compounds [2, 4] have been reported, but the anticancer effects of* Hyrtios* sp. have not been reported. Herein,* Hyrtios* sp. extract was investigated for anticancer activity in human colorectal carcinoma RKO cells with different p53 status.

## 2. Materials and Methods

### 2.1. Specimen Preparation

Marin sponge specimens were collected by hand with scuba equipment at Chuuk state, Federated States of Micronesia, in October, 2010. Freshly collected specimens were washed by sterilized artificial sea water three times, immediately frozen, and stored at −20°C until use. Lyophilized specimens were extracted with methanol (3 × 3 L) as previous study [7]. All of the samples were provided from KIOST (Korea Institute of Ocean Science & Technology). The extracts of specimens (10 mg) were dissolved in sterile distilled water (the final concentration, 50 mg/mL). Aliquots of samples were stored at −20°C until use.

### 2.2. Cells and Treatment

Human colorectal carcinoma RKO (CRL-2577) and RKO-E6 (CRL-2578) cells (ATCC, Manassas, VA) were cultured in Dulbecco's modified Eagle's medium (DMEM, GenDEPOT) supplemented with 10% fetal bovine serum (GenDEPOT) and 1% penicillin/streptomycin (GenDEPOT) in a humidified 5% CO_2_ incubator. Cells used for the assays were in exponential growth phase. The samples were treated to the cell culture for 24 h or 48 h.

### 2.3. Cell Cytotoxicity

Cell cytotoxicity was examined by Cell Counting Kit-8 (CCK-8, DOJINDO, Japan). Briefly, cells were seeded in 96-well plates at a density of 3 × 10^3^ cells/well. After incubation for 24 h, cells were treated with sponge samples for 24 h or 48 h. CCK-8 reagent (10 *μ*L) was added to each well and incubated for 3 h at 37°C. Absorbance at 450 nm was determined through microplate reader (Infinite M200 PRO, TECAN, Austria).

### 2.4. Cellular Morphology

Cells were seeded in 6-well plate at 4–6 × 10^4^ cells/well and treated with* Hyrtios* sp. extract (80 *μ*g/mL) for 48 h. Cells were observed under light microscopy (×40 and ×400) (Nikon eclipse TS100, Japan).

### 2.5. Western Blot Analysis

Cells were seeded in 6-well plate at a density of 4~6 × 10^4^ cells/well. Samples were treated to each well and incubated for 24 h or 48 h. Cells were harvested and lysed in RIPA buffer (GenDEPOT) with protease inhibitors (Xpert protease inhibitor cocktail solution, GenDEPOT) and phosphatase inhibitors (Xpert phosphatase inhibitor cocktail solution, GenDEPOT). Cell lysates were boiled in 5× sample buffer and separated by 10% SDS-PAGE. Proteins were transferred onto PVDF membranes (Millipore) using a semidry electroblotter (Peqlab, Germany). Membranes were blocked with 5% skim milk in TBST (50 mM Tris-HCl pH 7.4, 150 mM NaCl, 0.1% Tween 20) and incubated sequentially with primary antibodies at 4°C, overnight. After washing membranes with TBST, the membranes reincubated with secondary antibody at room temperature for 3 hr. Immunoreactive protein was visualized using ECL reagents and developed with X-ray film. Antibodies and ratios at which they were used were p53, p21 (1 : 2000, Millipore), c-Jun N-terminal kinase (JNK, 1 : 500, Santa Cruz Biotechnology), *β*-actin (1 : 5000, Sigma-aldrich), and anti-mouse IgG (H+L) horseradish peroxidase conjugate and anti-rabbit IgG (H+L) horseradish peroxidase conjugate (1 : 3000, Bio-Rad).

### 2.6. Apoptosis

Cells were seeded in 6-well plate and treated as described for western blots. After incubation, cells were stained with Annexin V-FITC (Nexcelom Bioscience LLC) and propidium iodide (PI) solution. Apoptosis was detected using a Cellometer (Nexcelom Bioscience LLC).

## 3. Results and Discussion

### 3.1. Cytotoxicity of* Hyrtios* sp. Extract in RKO and RKO-E6

**Figure 2:**
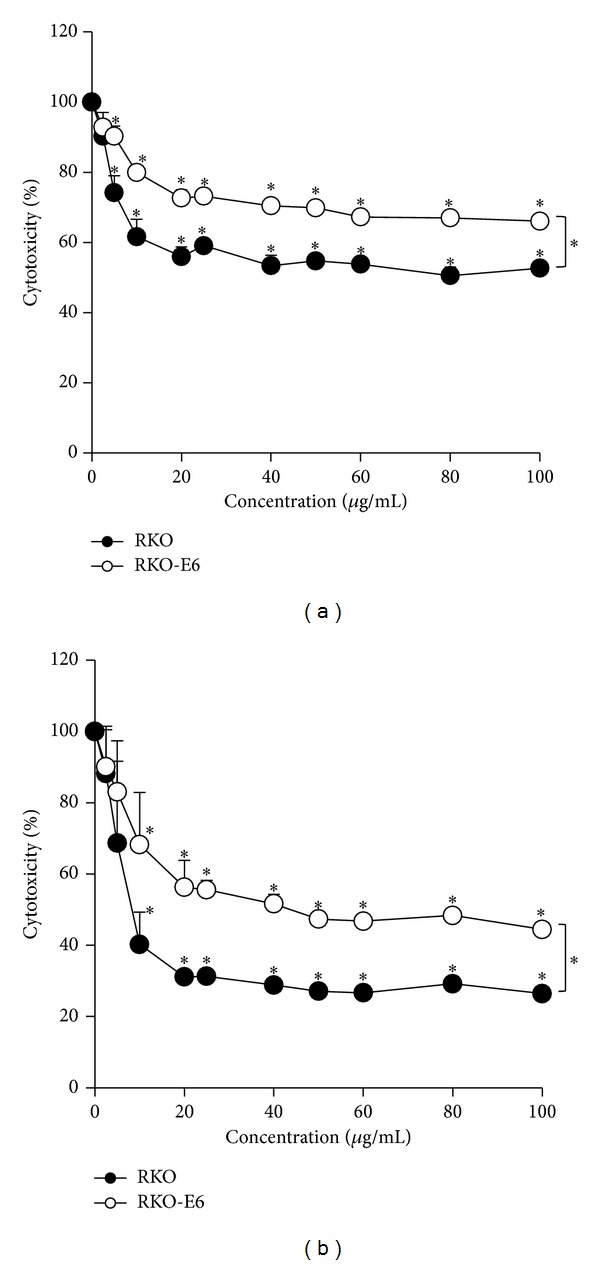
Inhibition of cell viability by* Hyrtios* sp. in RKO and RKO-E6 cells. Cells were treated with* Hyrtios* sp. extract and incubated for 24 h (a) and 48 h (b). Cytotoxicity was determined by CCK-8 assay. Data represent mean ± standard deviations (*n* = 7), *t*-test (*P < 0.001).

To evaluate cytotoxicity to cells with different p53 status, serially diluted samples of* Hyrtios* sp. extracts were treated to RKO and RKO-E6 cells for 24 h and 48 h. As shown in Figure 2(a), cytotoxicity slightly increased in both RKO and RKO-E6 cells.* Hyrtios* sp. extract (100 *μ*g/mL) inhibited 52.7 ± 0.9% viability of RKO cells and 66 ± 0.9% viability of RKO-E6 cells.* Hyrtios* sp. extract (100 *μ*g/mL) for 48 h inhibited 26.5 ± 0.7% and 44.5 ± 1% of cell viability in RKO and RKO-E6 cells, respectively (Figure 2(b)).* Hyrtios* sp. extract increased cytotoxicity time-dependently for RKO cells and RKO-E6 cells. The result showed that RKO cells were more sensitive than RKO-E6 cells to* Hyrtios* sp. extracts and indicated that the anticancer effects of* Hyrtios* sp. were different for RKO and RKO-E6 cells depending on their p53 status.

### 3.2. *Hyrtios* sp. Extract-Induced Mitotic Catastrophe

**Figure 3:**
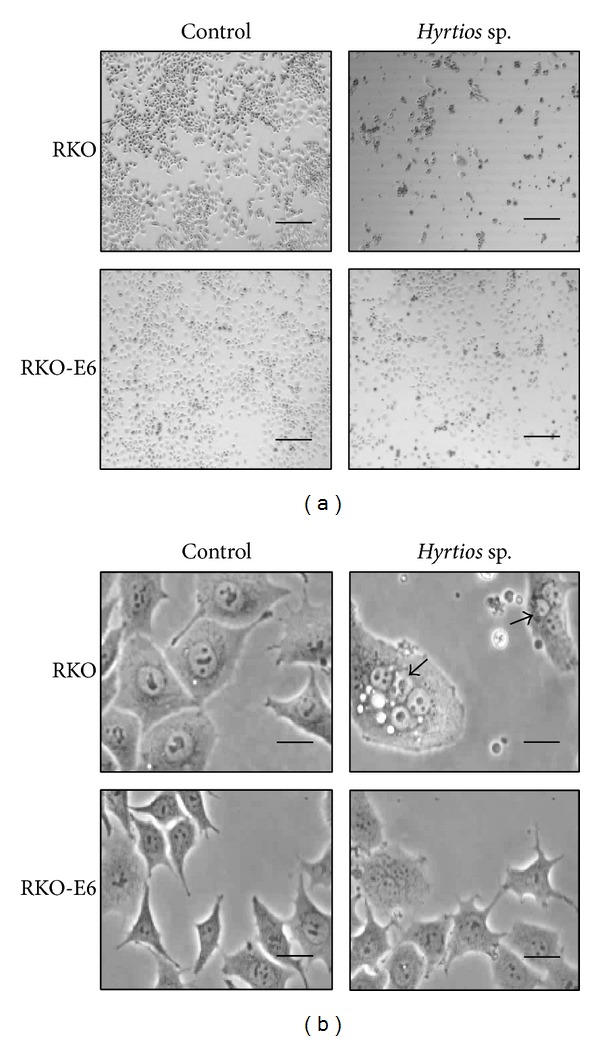
Change in cellular morphology after treatment with* Hyrtios* sp. extracts. Cells were treated with* Hyrtios *sp. extract (80 *μ*g/mL) and incubated for 48 h. (a) Scale bar = 500 *μ*m. (b) Scale bar = 50 *μ*m.

To investigate cell death induced by* Hyrtios* sp. extracts, cellular morphology was observed. RKO cells had fewer cells than RKO-E6 cells after treatment with* Hyrtios* sp. extracts (Figure 3(a)) consistent with cytotoxicity results. In particular, RKO cells treated with* Hyrtios* sp. extracts exhibited multinucleation and increased cell size, indications of mitotic catastrophe (Figure 3(b),* upper right arrow*).* Hyrtios* sp. might trigger the cell death induced by DNA damage.

### 3.3. Changes in p53, p21, and JNK Levels after Treatment with* Hyrtios* sp. Extract

**Figure 4:**
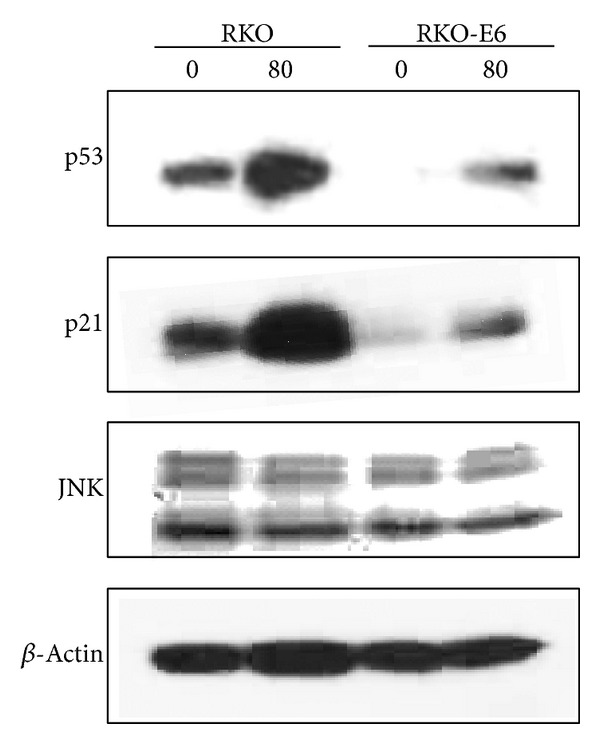
Expression of p53, p21, and JNK. Cells were treated with* Hyrtios* sp. extract (80 *μ*g/mL) and incubated for 48 h. Protein expression was determined by western blots according to “Section 2”.

To examine differences in cellular mechanisms between RKO and RKO-E6 cells, protein levels were determined by western blots. Expression of p53 protein was detected in RKO cells but not RKO-E6 cells (Figure 4). RKO cells treated with* Hyrtios* sp. extracts showed an increase of p53 protein levels and also increased expression of p21 protein which is encoded by a p53 target gene. In addition, p21 protein also increased in RKO-E6 cells treated with* Hyrtios* sp. extracts. JNK protein expression was slightly reduced in RKO and RKO-E6 cells (Figure 4). These results indicated that RKO cells exposed to* Hyrtios* sp. extracts induced cell death by increase of p53 protein, which activated the expression of p21 protein for 24 h (not shown) and 48 h, consistent with previous studies [8–10]. Surprisingly, RKO-E6 cells, which lack functional p53, showed slightly induced p53 protein expression after treatment with* Hyrtios* sp. extracts. However, RKO-E6 cells treated with* Hyrtios* sp. extracts inhibited cell growth through induction of p21 protein* via* a p53-independent pathway. These results suggested that suppression of JNK protein might be involved in p21 protein expression as previous study [11]. The JNK pathway is involved in phosphorylation of c-Jun [12] and p53 [13] and regulation of cell growth [14]. As reported by Potapova [15], inhibition of JNK reduced the growth of p53-deficient cells and induced p21 protein expression. Our results were consistent with this study [15].

### 3.4. Induction of Apoptosis by* Hyrtios* sp. Extract

**Figure 5:**
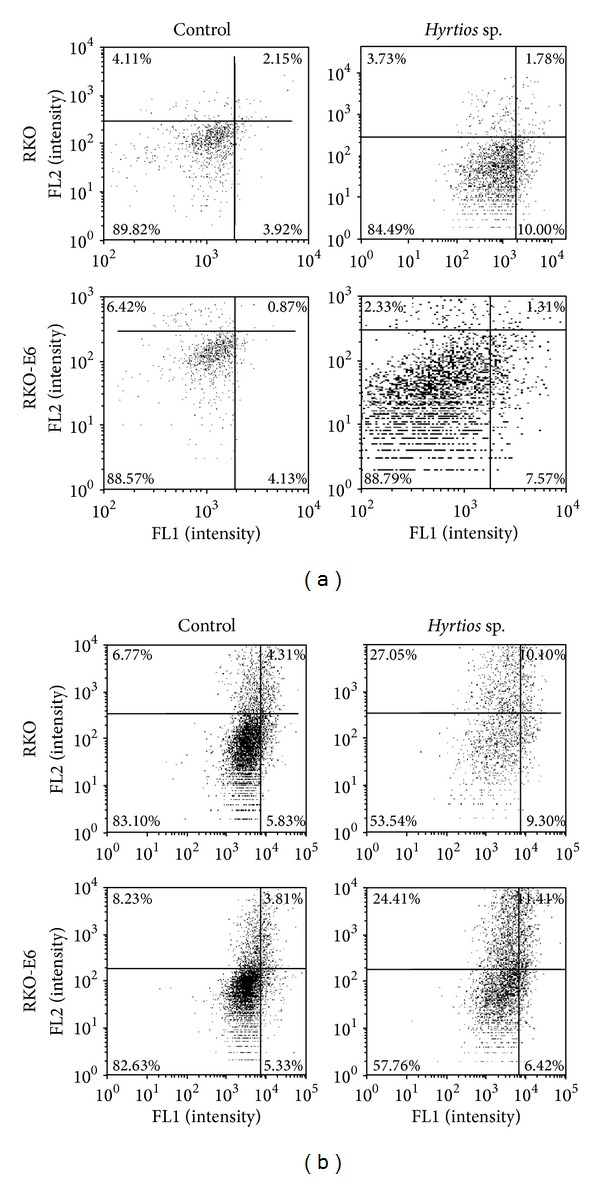
*Hyrtios* sp. extracts induced apoptosis. Cells were treated with* Hyrtios* sp. extract (80 *μ*g/mL) and incubated for 24 h (a) or 48 h (b). Apoptotic cells were detected by Annexin V and PI staining.

To verify cellular apoptosis, we used Annexin V and PI staining to investigate RKO and RKO-E6 cells treated with* Hyrtios* sp. extracts. As shown in Figure 5, apoptotic cells were detected similarly in RKO and RKO-E6 cells without treatment of* Hyrtios *sp. extracts.* Hyrtios* sp. extracts time-dependently decreased live cells and increased apoptotic cell death. RKO cells treated with* Hyrtios* sp. extract showed more apoptotic cells than RKO-E6 cells treated with* Hyrtios* sp. Extract. Therefore,* Hyrtios* sp. extracts induced more apoptosis in RKO cells than in RKO-E6 cells.

## 4. Conclusions

In this study, we investigated anticancer effects of* Hyrtios* sp. extract on p53 wild-type RKO cells and p53-deficient RKO-E6 cells. Our results indicated that* Hyrtios* sp. extract suppressed cell growth in both cell lines, but RKO cells, which have functional p53, were more sensitive to the cytotoxic effects of* Hyrtios* sp. extract than RKO-E6 cells. The anticancer effect of* Hyrtios* sp. extract was induced* via* activation of the p53 pathway in RKO cells and suppression of the JNK pathway in RKO-E6 cells.* Hyrtios* sp. is a natural marine sponge that could be a good candidate for cancer treatments. In a further study, the bioactive components of* Hyrtios* sp. will be investigated.
